# Significant Decrease in Scientific Performance after Completing Habilitation as an Academic Milestone: A Bibliometric Analysis of 742 Web of Science Profiles with Focus on Orthopedic and Trauma Surgeons

**DOI:** 10.1055/a-2658-0605

**Published:** 2025-08-21

**Authors:** Sam Razaeian, Julia Hoffmann, Emmanouil Liodakis, Marcus Örgel

**Affiliations:** 1 9379 Department of Trauma, Hand and Reconstructive Surgery, Saarland University, Homburg, Germany; 2 9177 Clinic for Trauma Surgery, Hannover Medical School, Hannover, Germany

**Keywords:** scientific performance, citation, normalized citation percentile, h-index, habilitation, wissenschaftliche Leistung, Zitation, normalized citation percentile, h-Index, Habilitation

## Abstract

**Purpose:**

Habilitation is a procedure by which one of the highest university degrees is achieved in the field of medicine in Germany. We hypothesize that this academic milestone represents an incentive for scientific productivity that drops off once a scientist has reached this career steep. This study aims to compare scientific performance of German scientists before and after completing this milestone with special focus on orthopedic surgeons and traumatologists (O&T).

**Methods:**

Scientists who had completed their habilitation in human medicine were researched from public announcements in the period Jan–Dec 2018. The periods Jan 2016 to Dec 2018 and Jan 2020 to Dec 2022 were defined as pre- and post-habilitation phases, respectively. Scientific performance was calculated using normalized citation percentiles (NCPs) from author records in Web of Science. Association between sex, subject area, and change in performance were analyzed.

**Results:**

NCP values of 742 scientists were analyzed showing a significant decrease after completing habilitation ( *p* < 0.001). This applied to men and women ( *p* = 0.015, *p* = 0.003) and non-surgical disciplines ( *p* = 0.001), while surgical disciplines such as O&T only demonstrated a statistically non-significant decrease. Interestingly, women showed an increase in performance after habilitation in this male-dominated discipline at only 4.5% (2) females compared to males. Most scientists in the population experienced a decline in performance (53.9% [400]). This drop amounted to over 50% in 35.5% (142) of these cases. No association was found regarding gender or subject area.

**Conclusions:**

Scientific performance seems to be incentive-dependent and significantly decreases after completing a career milestone in Germany. This decline is not statistically significant in O&T; women, who are strongly underrepresented, even show an increase in performance.

## Zusammenfassung

**Hintergrund:**

Die Habilitation ist ein Verfahren, mit dem in Deutschland einer der höchsten akademischen Titel im Bereich der Humanmedizin erreicht wird. Wir vermuten, dass dieser Meilenstein einen Anreiz für die wissenschaftliche Performance darstellt, die nach Erreichen dieser Karrierestufe nachlässt. Ziel dieser Studie ist es, die wissenschaftliche Performance deutscher Wissenschaftler vor und nach Erreichen dieses Meilensteins zu vergleichen; wurde ein besonderer Fokus auf Orthopäden und Unfallchirurgen (O&U) gelegt.

**Methoden:**

Wissenschaftlerinnen und Wissenschaftler der Humanmedizin mit abgeschlossener Habilitation wurden aus öffentlichen Bekanntmachungen zwischen 01/2018 und 12/2018 recherchiert. Die Zeiträume zwischen 01/2016 und 12/2018 sowie zwischen 01/2020 und 12/2022 wurden als Prä- und Posthabilitationsphasen definiert. Die wissenschaftliche Performance wurde anhand von normalisierten Zitationsperzentilen (NCPs) aus Autorenprofilen von Web of Science ermittelt. Ein möglicher Zusammenhang zwischen Geschlecht, Fachgebiet und Leistungsveränderung wurde ebenfalls analysiert.

**Ergebnisse:**

NCP-Werte von 742 Wissenschaftlern zeigten einen hochsignifikanten Abfall nach Abschluss der Habilitation ( *p* < 0,001). Dies galt für Männer und Frauen ( *p* = 0,015; *p* = 0,003) sowie für nicht chirurgische Disziplinen ( *p* = 0,001), während chirurgische Disziplinen wie O&U nur einen statistisch nicht signifikanten Rückgang aufwiesen. Interessanterweise zeigten Frauen im Gegensatz zu Männern eine Perfomancesteigerung nach der Habilitation in dieser männerdominierten Fachdisziplin mit nur 4,5% (2) Frauen. Bei den meisten Wissenschaftlern kam es zu einem Performanceabfall (53,9% [400]). In 35,5% (142) der Fälle betrug dieser Abfall mehr als 50 %. Ein Zusammenhang mit dem Geschlecht oder dem Fachgebiet konnte nicht festgestellt werden.

**Schlussfolgerung:**

Die wissenschaftliche Performance scheint anreizabhängig zu sein und nimmt nach Erreichen eines Karrieremeilensteins in Deutschland deutlich ab. Im Bereich O&U ist dieser Rückgang statistisch nicht signifikant; Frauen, die in diesem Bereich stark unterrepräsentiert sind, zeigen sogar einen Anstieg ihrer Performance.

## Introduction

Habilitation is a procedure by which one of the highest university degrees “PD” or “Priv.-Doz.” (Privatdozent) is achieved in the field of medicine in Germany 1 2 3 . This degree is an additional qualification at a higher level than the German doctoral degree, and it is usually a criterion for a full professorship within the German university system 1 2 3 4 . Therefore, habilitation represents a milestone in an academic career. A habilitation candidate must fulfill a university’s set criteria of excellence in research, teaching, and education over a long period 1 2 3 4 . Habilitation is awarded following a public lecture that the candidate delivers after the habilitation thesis has been accepted. Habilitation effectively includes the venia legendi (Latin: “permission to lecture”). The term habilitation is derived from the Latin term “habilis” (having sufficient ability to do or to conduct, being skillful) 5 . Consequently, it could be assumed that academic performance would increase or at least remain at the same high level once this higher qualification has been obtained if science is understood as an endogenous progress in which new, more meaningful findings are based on previous accumulated knowledge 6 . Recent evidence shows declining research productivity worldwide 6 . The volume of research papers published has skyrocketed over the past few decades, but the papers have become less novel compared to prior work 6 . The reasons for this phenomenon are unclear 6 7 .

We hypothesize that habilitation is a career milestone within the German university system, representing an incentive for scientific productivity that falls off once the scientist has achieved the object of this incentive.

This study aims to compare scientific performance among scientists of German universities using bibliometrics before and after completing their academic habilitation milestone.

This study has been authorized by the local ethical committee and was carried out in accordance with the Ethical standards of the 1964 Declaration of Helsinki as updated in 2004.

Scientific performance of all habilitation candidates of German universities in the field of human medicine from 2018 was compared before and after they completed their habilitation. The candidates were researched from announcements of a publicly accessible German university news portal and the biggest national medical journal 8 9 . All candidates from the field of human medicine who had been announced as having completed their habilitation between January 2018 and December 2018 were included.

A three-year period between January 2016 and December 2018 was defined as the period before habilitation (prehab phase). The period after the habilitation (posthab phase) was defined as one year after the year of habilitation (2018) between January 2020 and December 2022. A one-year gap (2019) up to the habilitation year was deliberately chosen. This year was not evaluated to prevent lingering achievements during the prehab phase from distorting results from the posthab phase.

Scientific performance was calculated using established bibliometric parameters from author records in the Web of Science (WoS) 10 . Access took place between November 2023 and December 2023. The primary performance indicator was defined as the normalized citation percentile (NCP). NCPs are part of author impact beamplots 11 . An NCP indicates how a publication has performed relative to its peers, and how performance has changed over time, and therefore serves a normalized performance indicator 12 . NCP values are determined by comparing citations for a single publication to citation counts for all publications in the same year, subject category, and article type, and calculating the percentage of papers at each level of citation. Higher NCP values indicate better performance 12 . For example, a publication with a percentile of value of 99 is in the top 1% of most cited publications in the same article type, year, and subject category 12 . Changes in NCP values of 2016 and 2017 were compared against values of 2020 and 2021. The shortened period was selected because NCPs are not offered for the current and previous analysis year, and analysis was only possible up to 2021 at the time of the study design.

The number of publications, number of citations, and self-citations before and after habilitation were used as secondary performance indicators.

Sex of candidates was determined based on their first names. In addition to the affiliated university, the habilitation subject was categorized into surgical and non-surgical. The profile with the most entries was evaluated for each person with more than one author profile in the WoS. The number of publications, citations, and self-citations as well as h-index of the entire career period were also recorded.

### Statistical analysis

Descriptive statistics were calculated. Interval and ordinal dependent variables were compared using the Mann-Whitney U test. NCP values from the two years in the prehab and posthab phases were pooled for group comparison. To calculate percentage change of NCP values, ordinal scaled NCPs in the two years of the prehab and posthab phases were first summed together. The NCP was raised from 0 to 1 for individuals without publications that year to avoid division by zero in the percentage calculation. Percentage change in NCP between the two phases was classified as follows: ≤−50%: strong; ≤−25%: moderate; < 0%: slight; 0%: no change; > 0%: slight; > 25%: moderate; > 50%: strong. Chi-squared test and Fisher’s exact test were used in analyzing the association between sex, subject area, and change in scientific performance. For this purpose, percentage change in NCP was calculated as categorial variable (decrease: < 0%; increase: ≥ 0%).

The confidence level (CI) was set at 95% ( *p* < 0.05). Data analysis was performed using SPSS 26.0 (IBM, Armonk, New York) and Microsoft Excel 2019 (Microsoft Corporation, Redmond, Washington).

## Results

The analysis encompassed 742 habilitation candidates announced from 36 universities. Table 1 lists all the cities where the universities were located and the frequency distribution of scientists.

**Table:** **Table 1** List of associated cities of included universities, and frequency distribution of scientists. Data are from Web of Science, provided by Clarivate. Web of Science and Clarivate are trademarks of their respective owners and used herein with permission.

University/city	*n*	%
Aachen	30	4
Bochum	12	1.6
Bonn	32	4.3
Berlin Charite	38	5.1
Dresden	22	3
Düsseldorf	19	2.6
Erlangen	32	4.3
Essen	27	3.6
Frankfurt am Main	12	1.6
Freiburg	34	4.6
Gießen	12	1.6
Göttingen	16	2.2
Greifswald	2	0.3
Halle-Wittenberg	4	0.5
Hamburg	42	5.7
Hanover	37	5
Heidelberg	3	0.4
Homburg	11	1.5
Jena	14	1.9
Cologne	23	3.1
Leipzig	20	2.7
Magdeburg	7	0.9
Mainz	12	1.6
Mannheim	21	2.8
Marburg	12	1.6
Technical University of Munich	39	5.3
Ludwig Maximilian University of Munich	47	6.3
Münster	30	4
Regensburg	32	4.3
Rostock	4	0.5
Kiel	13	1.8
Lübeck	13	1.8
Tübingen	32	4.3
Ulm	21	2.8
Witten/Herdecke	4	0.5
Würzburg	13	1.8

Men were represented more often than women by a majority of over two thirds (70% [519]). In surgical disciplines, this distribution was much more divergent with a significant association between sex, and subject area ( *p* = 0.001; Table 2 ).

**Table:** **Table 2** Association between sex, subject area, and change in scientific performance.

	Change in NCP ^1^		NCP median (IQR)				*p* value²
	Decrease, *n* (%)	Increase, *n* (%)	2016	2017	2020	2021	
** Surgical subject, *n* (%) **							
Male, 116 (81.1)	62 (53.4)	54 (46.6)	44 (35)	46 (35)	46 (36)	40 (45)	0.2
Female, 27 (18.9)	14 (51.9)	13 (48.1)	59 (46)	48 (42)	50 (28)	45 (67)	0.4
** Non-surgical subject, *n* (%) **							
Male, 403 (67.3)	215 (53.3)	188 (46.7)	54 (44)	49 (41)	50 (52)	47 (58)	**0.036**
Female, 196 (32.7)	109 (55.6)	87 (44.4)	54 (53)	59 (46)	49 (66)	47 (66)	**0.005**
^1^ Percentage change in NCP was calculated as categorial variable for chi-squared test, ^2^ differences in pooled NCPs between 2016 and 2017 versus 2020 and 2021; bold *p* value indicates statistically significant difference between groups ( *p* < 0.05), NCP: normalized citation percentile, IQR: interquartile range. Data are from Web of Science, provided by Clarivate. Web of Science and Clarivate are trademarks of their respective owners and used herein with permission.							

Scientific performance defined by NCP values decreased significantly after completing habilitation ( *p* < 0.001; Table 3 ). This applied to both men and women ( *p* = 0.015, *p* = 0.003; Table 4 ), but without any significant association between gender, subject area, and change in performance (χ² [1, *n* = 742] = 0.3, *p* = 1; Table 2 ).

**Table:** **Table 3** Mean ± standard deviation (SD), median and interquartile range (IQR) of analyzed scientometric parameters.

	Publications, mean (±SD)	Citations, mean (±SD)	Self-citations, mean (±SD)	h-index, mean (±SD)	NCP median (IQR)				*p* value ^2^
					2016	2017	2020	2021	
** Subjects, *n* (%) **									
Total, 742 (100)	35.1 (33.7)	411.3 (660)	6.5 (9.5)	10 (6.2)	52 (44)	50 (42)	48 (53)	46 (57)	**< 0.001**
Surgical, 143 (19.3)	26.6 (18.5)	252.3 (235.8)	4.2 (4.6)	8.14 (4.1)	46 (37)	47 (36)	47 (35)	40 (51)	0.1
Non-surgical, 599 (80.7)	37.1 (36.1)	449.2 (720.4)	7.1 (10.3)	10.5 (6.1)	54 (45)	51 (43)	50 (56)	47 (58)	**0.001**
*p* value ^1^	**0.016**	**0.008**	**0.005**	**< 0.001**	0.1	0.052	0.1	0.1	
**Phase (years)**									
Prehab (2016–2018)	14.6 (13.4)	58.2 (104.9)	3 (4.2)	n.m.					
Posthab (2020–2022)	15.9 (18.4)	92.4 (241.2)	3.6 (6.7)	n.m.					
*p* value ^1^	0.23	**0.03**	0.16						
NCP: Normalized citation percentile, n.m.: not measured, ^1^ mean difference of subgroups, ^2^ differences in pooled NCPs between 2016 and 2017 versus 2020 and 2021, bold *p* value indicates statistically significant difference between groups ( *p* < 0.05). Data are from Web of Science, provided by Clarivate. Web of Science and Clarivate are trademarks of their respective owners and used herein with permission.									

However, women in surgical disciplines had lower rates of decline in performance than women in non-surgical subject areas (51.9% vs. 55.6%; Table 2 ).

**Fig. 1:**
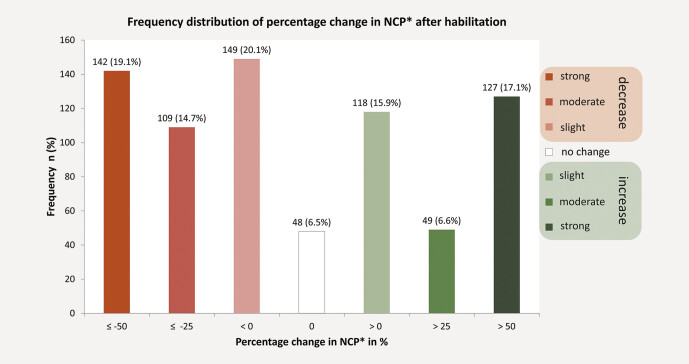
Frequency distribution of percentage change in NCP after completing habilitation. Data are from Web of Science, provided by Clarivate. Web of Science and Clarivate are trademarks of their respective owners and used herein with permission.

Most scientists experienced a decline in performance (53.9% [400]). This decline was moderate to severe in a third of the total study population ( Fig. 1 ). Performance increased in 39.6% (294) of the cases and remained at the same level in 6.5% (48) of the cases. Fig. 1 shows frequency distribution of percentage change in NCP after completing habilitation.

Women published significantly less than men in the prehab phase and in total ( *p* = 0.004, *p* = 0.034; Table 4 ). Nevertheless, they had similar mean values regarding number of citations, self-citations, and h-index, and they tended to be superior in their performance to the men in the prehab phase considering NCP median values. Their performance was even significantly higher in 2017, immediately before habilitation ( *p* = 0.003). This advance diminished in the posthab phase, and their level of performance was comparable to that of men ( Table 4 ). Secondary performance indicators – number of publications, number of self-citations – did not change significantly, but the absolute number of citations increased significantly in the posthab phase ( *p* = 0.03; Table 4 ).

**Table:** **Table 4** Mean ± standard deviation (SD), median and interquartile range (IQR) of analyzed scientometric parameters regarding gender differences.

	Publications, mean (±SD)	Citations, mean (±SD)	Self-citations, mean (±SD)	h-index, mean (±SD)	NCP median (IQR)				*p* value ^2^
					2016	2017	2020	2021	
** Sex *n* (%) **									
Male 519 (70)	37 (35.8)	407.5 (623.9)	6.8 (10.1)	10.1 (6.3)					
Female 223 (30)	30.6 (27.8)	420.0 (738.4)	5.9 (8)	9.9 (6.2)					
*p* value ^1^	**0.034**	0.7	0.3	0.8					
**Phase (years)**									
Prehab (2016–2018)									
Male	15.5 (14.1)	58.61 (108)	3.11 (4.4)	n.m.	52 (42)	49 (40)			
Female	12.5 (11.3)	57.15 (97.6)	2.68 (3.5)	n.m.	54 (45)	56 (45)			
*p* value ^1^	**0.004**	0.4	0.4		0.4	**0.003**			
Posthab (2020–2022)									
Male	16.59 (19.6)	93.63 (269.7)	3.7 (7)	n.m.			48 (48)	46 (55)	**0.015**
Female	14.12 (15)	89.47 (156.6)	3.2 (6)	n.m.			49 (63)	46 (67)	**0.003**
*p* value ^1^	*0.4*	*0.5*	*0.2*				*0.3*	*0.9*	
NCP: Normalized citation percentile, n.m.: not measured, ^1^ mean difference of subgroups, ^2^ differences in pooled NCPs between 2016 and 2017 versus 2020 and 2021, bold *p* value indicates statistically significant difference between groups ( *p* < 0.05). Data are from Web of Science, provided by Clarivate. Web of Science and Clarivate are trademarks of their respective owners and used herein with permission.									

### Orthopedic surgeons and traumatologists (O&T)

NCPs of 44 scientists were examined and showed a statistically non-significant decrease in performance after habilitation ( Table 5 ). This decline was moderate to severe in 31.8% (14) of this subgroup.

**Table:** **Table 5** Mean ± standard deviation (SD), median and interquartile range (IQR) of orthopedic surgeons, and traumatologists regarding differences by sex.

	Publications, mean (±SD)	Citations, mean (±SD)	Self-citations, mean (±SD)	h-index, mean (±SD)	NCP median (IQR)				*p* value ^2^
					2016	2017	2020	2021	
** Total, *n* (%) **									
44 (100)	30.5 (17.4)	269.8 (157.8)	4.9 (4.5)	8.9 (3.1)	48 (27)	45 (35)	49 (29)	42.5 (39)	0.8
** Sex, *n* (%) **									
Male, 42 (95.5)	31 (17.6)	269.9 (158.5)	5 (4.5)	8.9 (3.1)					
Female, 2 (4.5)	20 (8.5)	269 (202.2)	3.5 (2.12)	10 (2.8)					
*p* value ^1^	0.4	0.9	0.8	0.5					
**Phase (years)**									
Prehab (2016–2018)									
Male	13.8 (7.9)	31.5 (22.5)	2.8 (2.5)	n.m.	48 (26)	45 (38)			
Female	12 (5.7)	42.5 (46)	3 (2.8)	n.m.	60.5 (n.a.)	41.5 (n.a.)			
*p value* ^1^	*0.9*	*0.8*	*0.8*		*0.4*	*0.08*			
Posthab (2020–2022)									
Male	13.9 (10.2)	38.8 (31.8)	2.2 (3)	n.m.			48.5 (26)	41.5 (37)	1
Female	7 (2.8)	37 (26.9)	0.5 (0.7)	n.m.			74.5 (n.a.)	61.5 (n.a.)	0.4
*p* value ^1^	0.4	0.9	0.4				0.1	0.2	
NCP: Normalized citation percentile, n.m.: not measured, n.a.: not applicable, ^1^ mean difference of subgroups, ^2^ differences in pooled NCPs between 2016 and 2017 versus 2020 and 2021, bold *p* value indicates statistically significant difference between groups ( *p* < 0.05). Data are from Web of Science, provided by Clarivate. Web of Science and Clarivate are trademarks of their respective owners and used herein with permission.									

Women were strongly underrepresented in this male-dominated discipline (4.5%, 2). However, similar to the total cohort, women published less than men but demonstrated nearly equal mean values regarding number of citations, and h-index. In addition, they tended to be superior in their performance to the men in both phases regarding NCP median values. In contrast to men, their performance even tended to increase after habilitation ( Table 5 ).

## Discussion

This is the first study to investigate scientific performance in the field of medicine among scientists of German universities with particular focus on their performance before and after finishing habilitation that functions as an academic milestone.

Our principal finding was that most scientists in Germany experienced a significant decline in scientific performance measured by an established bibliometric parameter after reaching that milestone. This decline in performance was moderate to severe in a third of the study population ( Fig. 1 ). The primary performance indicator measured by using the normalized citation percentile decreased significantly, whereas secondary performance indicators, such as the number of publications and absolute number of citations, remained almost the same or increased significantly.

If science is understood as a progress in which new, more meaningful findings are based on previous ones 6 7 , and completing the habilitation is understood as just one milestone on this path that proves the ability to conduct independent research, then this observation is surprising. One would expect that academic performance would at least remain the same or increase further after habilitation. However, the opposite effect as observed – an decrease – supports our assumption that habilitation might be a career milestone within the German university system representing an incentive for scientific productivity that declines once a scientist has achieved the object of this incentive. These individuals might have primarily been interested in obtaining an academic title and could have influenced the results by representing a “low hanging fruits” mentality 6 7 . However, other reasons are also conceivable. Personal circumstances could have been responsible for a temporary focus on other aspects of both private and professional life after reaching this career milestone. A temporary reduction in performance to recover intellectual creativity after the strenuous, time-consuming habilitation phase is conceivable.

Our finding that women tend to lose performance slightly more than men could underline this assumption. Women are known to be exposed to greater hurdles in their academic careers 13 14 15 16 , and the far lower number of habilitation candidates may reflect the additional burden of family planning in the following years 14 15 16 . Interestingly, we observed that women have published significantly less than men. Nevertheless, their mean values regarding number of citations and h-index were similar, and their performance measured by NCPs even tended to be superior to men in the prehab phase, which might confirm that a high quantity (number of publications) does not necessarily guarantee scientific quality.

Women’s performance in the year 2017, immediately before habilitation, was even significantly higher. This advantage flattened out after finishing habilitation, reaching a performance level comparable to that of men.

Women were strongly underrepresented (4.5%, 2) in the male-dominated O&T discipline, and tended to be superior to the men in both phases. In contrast to the men, their performance even tended to increase after reaching the academic milestone. Our observation that women surgical disciplines have a lower rate of performance loss than in non-surgical subjects, where the proportion of women is higher, suggests that this outsider role may be responsible for the result. However, our data subgroups are too different in sample size to allow a valid interpretation of these gender differences.

This study has several strengths. In addition to the large sample size, one of the strengths is the usage of NCPs as a performance indicator instead of the widely known h-index 11 12 17 . This index characterizes a researcher’s publication and citation counts to a single number that depends on career length and discipline as citation counts accumulate over time at rates that vary between research fields 12 17 18 . Therefore, it does not provide valid comparability between individuals and is mathematically inconsistent 12 18 .

Bornmann and Marx of the Max Planck Society introduced and developed usage of NCP containing impact beamplots for bibliometric data. The Institute for Scientific Information (ISI) has promoted NCP containing beamplots as an alternative to the h-index as they contextualize a researcher’s article to make them suitable for comparison; this reduces the risk of reliance on a single-point metric that lacks context and nuance, and performance changes can be evaluated over the course of a researcher’s career 12 19 20 .

However, this study also has several limitations to consider. Firstly, the results are only transferable to a single country with a specific university system and only one discipline. Secondly, the results are limited by selection bias, as only a sample of scientists from 2018 was examined. Only scientists whose names had been publicly announced on the two accessed platforms could be included. Thirdly, the study period was short. Performance was examined just one year after the habilitation. It remains unclear whether and for how this decline would be observed over a longer examination period.

## Conclusion

Scientific performance seems to be incentive-dependent and significantly decreases after completing a career milestone in Germany, but without any significant association between gender, subject area, and change in performance. In O&T, this decline is not statistically significant; women, who are strongly underrepresented, even tend to increase their performance.
